# A Review on the Development of Earthquake Warning System Using Low-Cost Sensors in Taiwan

**DOI:** 10.3390/s21227649

**Published:** 2021-11-18

**Authors:** Yih-Min Wu, Himanshu Mittal

**Affiliations:** 1 Department of Geosciences, National Taiwan University, Taipei 10617, Taiwan; 2 Institute of Earth Sciences, Academia Sinica, Taipei 11529, Taiwan; 3 Research Center for Future Earth, National Taiwan University, Taipei 10617, Taiwan; 4 National Center for Seismology, Ministry of Earth Sciences, Government of India, New Delhi 110003, India; himanshu.mittal@gov.in

**Keywords:** P-Alert, earthquake early warning, MEMS, shakemaps

## Abstract

Seismic instrumentation for earthquake early warnings (EEWs) has improved significantly in the last few years, considering the station coverage, data quality, and the related applications. The official EEW system in Taiwan is operated by the Central Weather Bureau (CWB) and is responsible for issuing the regional warning for moderate-to-large earthquakes occurring in and around Taiwan. The low-cost micro-electro-mechanical system (MEMS)-based P-Alert EEW system is operational in Taiwan for on-site warnings and for producing shakemaps. Since 2010, this P-Alert system, installed by the National Taiwan University (NTU), has shown its importance during various earthquakes that caused damage in Taiwan. Although the system is capable of acting as a regional as well as an on-site warning system, it is particularly useful for on-site warning. Using real-time seismic signals, each P-Alert system can provide a 2–8 s-long warning time for the locations situated in the blind zone of the CWB regional warning system. The shakemaps plotted using this instrumentation help to assess the damage pattern and rupture directivity, a key feature in the risk mitigation process. These shakemaps are delivered to the intended users, including the disaster mitigation authorities, for possible relief purposes. Earlier, the network provided only peak ground acceleration (PGA) shakemaps, but has now been updated to include peak ground velocity (PGV), spectral acceleration ($S_{a}$) at different periods, and CWB intensity maps. The PGA and PGV shakemaps plotted using this network have proven helpful in establishing the fact that PGV is a better indicator of damage detection than PGA. This instrumentation is also useful in structural health-monitoring and estimating co-seismic deformations. Encouraged by the performance of the P-Alert network, more instruments are installed in Asia-Pacific countries.

## 1. Introduction

Every year, various natural hazards, including earthquakes, cause fatalities and property damage by affecting numerous people around the world. With advancements in technology and data processing speed, risk mitigation tools, such as earthquake early warnings (EEW), have emerged as life-saving guards in many earthquake-prone countries. The primary purpose of EEW is to detect an earthquake in the early stage, estimating the shaking intensity in the target regions, and to warn the users before experiencing the strong ground motion. Unlike other warning systems (typhoon, tsunami, volcano, flood, etc.), an hours- or minutes-long warning time is generally not possible. Despite this, the seconds-long warning achieved during EEW may be very helpful in saving the lives of human beings by allowing them to flee from buildings (if possible), or to take the proper shelter, or to move to a safer place within a building. Many countries, including Mexico [1,2], Japan [3], Taiwan [4,5], and South Korea [6], have developed EEW systems and are issuing warnings to the public and to authorities. Several other countries, namely, the United States [7], China [8], Turkey [9], Italy [10], and India [11,12], are in the process of developing and testing the EEW system [13].

The EEW system, in its principle form, takes information related to basic phases (P-wave and other phases) from the real-time seismic signals, performs the elementary calculations, and, if needed, issues a warning. The EEW system is not limited to major earthquakes only, but also targets smaller earthquakes, which may cause high shaking in local areas, as well. EEW systems are generally categorized as regional (network-based) and on-site (single station or network-based) systems. The regional system consists of several sensors placed around the fault/source, and seismic signals from these instruments are transferred continuously to the central station for processing [14,15,16,17,18]. The regional EEW system exploits the use of P-wave and some S-wave information [19] to estimate the location and magnitude of the earthquake and to predict the ground-shaking at farther distances using ground-motion prediction equations (GMPE). Once the earthquake is triggered using short-term averaging (STA), long-term averaging (LTA), or another algorithm, the initial few seconds of data (typically 3–5 s) after the P-wave’s arrival is used to perform the calculations. The regional EEW system generally takes about 10–15 s to detect an earthquake and issue a warning. By that time, the damaging S-waves reach some of the locations close to the epicenter and a warning is not possible. The areas without a warning are termed “blind zones” and may range around 40–60 km from the epicenter, depending upon how quickly an earthquake is located. The problem of the blind zone can be overcome by the on-site EEW system, under which a single station installed in the proximity of the target area will immediately sense the earthquake and issue the warning. This system will use the P-wave information and estimate the ground shaking using empirical scaling relationships. The on-site warning system is faster than the regional system and can provide early warnings to sites located in the near-source region. The accuracy of the estimation of earthquake parameters is moderate for an on-site EEW, as it is a single sensor-based or a small network-based system. The on-site EEW is functional in many countries, for example, in Japan [20], Bucharest, Romania [21], Istanbul [9], and Taiwan [22]. In Romania, a simple and effective EEW system is designed from the earthquakes in the Romanian Vranceazone, south-eastern Carpathians, and provides useful lead-times. The important parameter in EEW is the available time before the arrival of damaging S-waves’ or surface waves’ peak amplitude (called the lead-time). Based on the hypocentral distance between the source and target, the lead-time may be different using two approaches. Using the regional approach, a greater lead-time is achieved at larger distances; however, at smaller distances, on-site EEW systems are useful when the regional EEW fails.

**Figure 1:**
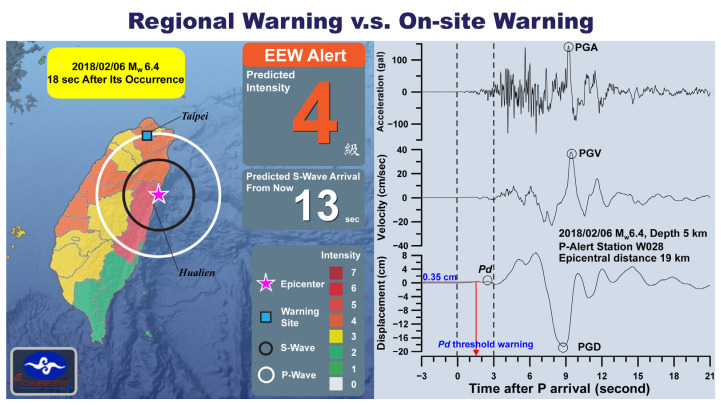
A comparison of time taken for the regional and on-site warnings. The left side shows that the CWB network took 18 s to issue a 13 s regional warning (lead-time) for Taipei City during the Hualien earthquake of 6 February 2018. The right side of the figure shows the $P_{d}$ estimation using the initial 3 s of the waveform at one of the stations of the P-Alert network, where the threshold value was achieved in 1.5 s.

Figure 1 depicts the difference in time taken for the issuance of the regional and on-site warnings. The left side shows the 13 s regional warning (lead-time) with intensity 4, issued by the Central Weather Bureau (CWB) network for Taipei City during the Hualien earthquake of 6 February 2018. The CWB network took 18 s to predict the intensity and issued the regional warning after the earthquake’s occurrence. By the time CWB issued the warning, the damaging S-waves reached the area encircled by the black line, where no warning was possible. However, the on-site warning network worked very well in this blind zone and issued the on-site warning (2–8 s). The working of the on-site EEW network during the Hualien earthquake is discussed by Wu et al. (2019) [15]. The right side of Figure 1 shows the peak amplitude of the vertical displacement, $P_{d}$, estimated using the initial 3 s of P waves at one of the stations of the P-Alert network located at an epicentral distance of 19 km. Based on the previous works [23,24], $P_{d}$ is the recommended parameter for on-site EEWs, where shaking corresponding to $P_{d}$ > 0.5 cm may cause damage. The threshold value $P_{d}$ ≥ 0.35 cm for issuing the warning is obtained at 1.5 s after the P arrival. It could have several seconds of lead-time before peak ground motion, which shows the efficiency of on-site warnings in the blind zones of regional warnings.

Building an efficient EEW system is a cost-effective process, as the stations should be densely distributed to detect and report the earthquake in minimal time. Generally, for a small area, EEW may be achieved by placing numerous instruments around seismogenic sources. A dense EEW system using traditional sensors is not feasible, especially in countries facing seismic hazards from a wide area. However, in recent years, low-cost sensors have emerged as an alternative to traditional sensors. The low-cost micro-electro-mechanical system (MEMS) sensors were introduced for EEW in 1990 [25] and are used by most of the countries working with EEW. Some countries have built their EEW network using these sensors only.

## 2. The EEW Systems in Taiwan

Taiwan is one of the countries that faces frequent seismic activities due to the ongoing subduction of the Philippine and Eurasian plates. Given the importance of property damage and economic loss, an EEW was conceptualized in Taiwan after the occurrence of the Hualien offshore $M_{w}$ 7.8 earthquake that caused extensive damage in the capital city Taipei in 1986, around 120 km away from the epicenter. S waves take more than 30 s to cover this distance. If a seismic system can detect an earthquake in the Hualien region within 20 s, then there could be a 10 s warning time for the Taipei metropolitan region. Thus, the implementation of the prototype EEW system by the CWB started in 1994 [26]. After several years of development, the CWB established a nationwide EEW system in 2002 [27,28]. To shorten the earthquake reporting time, the P-wave method is used in the CWB system [29,30,31]. Currently, the CWB system can regularly issue warnings within 20 s of an earthquake’s occurrence (Figure 1). The CWB system is in charge of providing earthquake alerts in Taiwan via text message through mobile phones, TVs, and direct broadcasting systems in schools [4,32]. This is a regional network and issues warning to places that are around 40–60 km away from the epicenter.

The other EEW system, run by the National Center for Research on Earthquake Engineering (NCREE), comprises 90 instruments installed in elementary schools and acts as an on-site and hybrid network [22]. Both of the networks discussed above function very well and the CWB system is capable enough to detect earthquakes and issue the warning. However, to study the fault mechanism in detail, and to issue the warning in the blind zone using the on-site mechanism, the instruments should be placed densely around the fault zone. Taiwan has many faults, inland and offshore, so many instruments are required to instrument the fault zones densely. As the traditional instruments are not feasible to place closely together, the National Taiwan University (NTU) installed the low-cost sensors to minimize the cost.

## 3. The P-Alert Sensors and EEW

**Figure 2:**
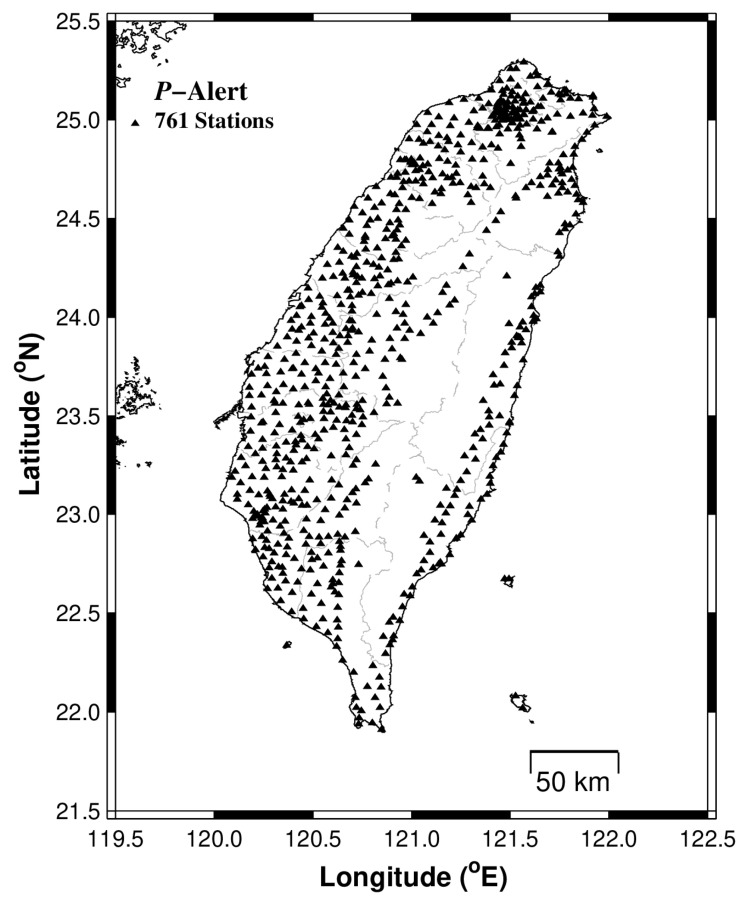
A map showing the distribution of P-Alert instruments in different parts of Taiwan. This dense array, consisting of 761 low-cost sensors, can act as an on-site, as well as a regional EEW system.

The research group at NTU worked in close association with a technology company in Taiwan for the development of a low-cost, MEMS-based, P-wave alert device named “P-Alert”. These MEMS-based sensors are embedded in a small housing and can record high-frequency, near-source ground-motion. The pilot project commenced in 2010 by installing 15 P-Alert devices in the Hualien part of the country [33]. The network showed its ability by detecting and recording earthquakes. Based on its performance in the Hualien region, the network was extended to other parts of the country. As of now, 761 P-Alert instruments have been installed under this network (Figure 2). With a threshold parameters algorithm embedded inside, the P-Alert sensors are suitable for on-site, as well as for regional warnings. Considering the proper logistics (continuous power supply and dedicated internet connection for data transfer) for the P-Alert installation, most of these instruments are installed in elementary schools on the ground or first floor. Each P-Alert device can record three-component data, having 16-bit resolution and ±2 g of full dynamic range. The sampling rate of all the instruments is set to be 100 Hz and the real-time three-component continuous data is transferred and processed continuously at the central receiving station placed at NTU and Academia Sinica.

For on-site warning, as per the algorithm embedded in the P-Alert instruments, the data received by each of the field instruments is continuously monitored for STA/LTA ratios, peak ground acceleration (PGA), and $P_{d}$ obtained by double-integrating the real-time data. The records are high-pass filtered after taking the integration. Once an earthquake has been declared using STA/LTA algorithm, the software will look for $P_{d}$ and PGA, using the initial few seconds of data (usually 3–4 s) after the P-wave arrival [23] as the warning. Once the threshold parameters are exceeded ($P_{d}$ ≥ 0.35 cm or PGA ≥ 80 gals), a warning is issued [34].

**Figure 3:**
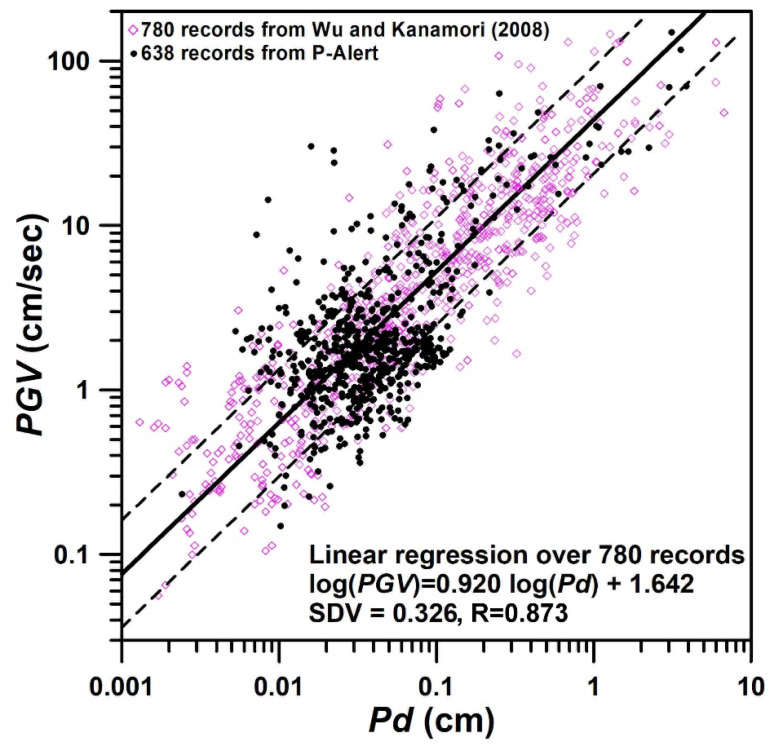
The relationship of $P_{d}$ with PGV by augmenting the 638 P-Alert records obtained during the 5 February 2016 Meinong earthquake and the 6 February 2018 Hualien earthquake with 780 records from Taiwan, Japan, and California via the strong motion instrument used by Wu and Kanamori (2008) [24].

Numerous works have been carried out for on-site warnings using the initial portion of the P-waves. Wu and Kanamori (2005b) [35] suggested the prediction of earthquake magnitude by using the inverse of the predominant period, $\tau_{c}$, from the initial 3 s of P-wave waveforms. Considering the trade-off between cost and data quality, the dynamic range of P-Alert sensors is less than that of traditional sensors. Thus, the calculation of traditional frequency-based parameters proposed earlier for EEW, such as $\tau_{c}$, may not be accurate. The same authors [23] established a regression relationship between $P_{d}$ and Peak Ground Velocity (PGV), and proposed that earthquakes may be damaging whenever $P_{d}\geq 0.5\;\mathrm{cm}$. Wu and Kanamori (2008) [24] also worked to predict PGV with $P_{d}$, using the various earthquakes recorded in Taiwan, Japan, and California (Figure 3). Using the regression carried out from data from different parts of the world, the $P_{d}$ parameter is considered one of the pioneering parameters for estimating shaking intensity.

To check the threshold value of $P_{d}$ in Taiwan, the relation of PGV and $P_{d}$ established by Wu et al. (2005a, 2008) [23,24] is validated using the P-Alert records. The 638 P-Alert records within the epicentral distance of 200 km, obtained during the 5 February 2016 $M_{w}$ 6.4 earthquake in Meinong and the 6 February 2018 $M_{w}$ 6.4 earthquake in Hualien, are augmented with 780 records from Taiwan, Japan, and California, via the strong motion instrument used by Wu and Kanamori (2008) [24]. By plotting the data recorded by the P-Alert instruments during the two earthquakes in Taiwan, the relationship is the same as the values from P-Alert records that lie within the original data of Wu and Kanamori (2008) [24], which shows that P-Alert records can be used successfully for $P_{d}$ calculation (Figure 3).

**Figure 4:**
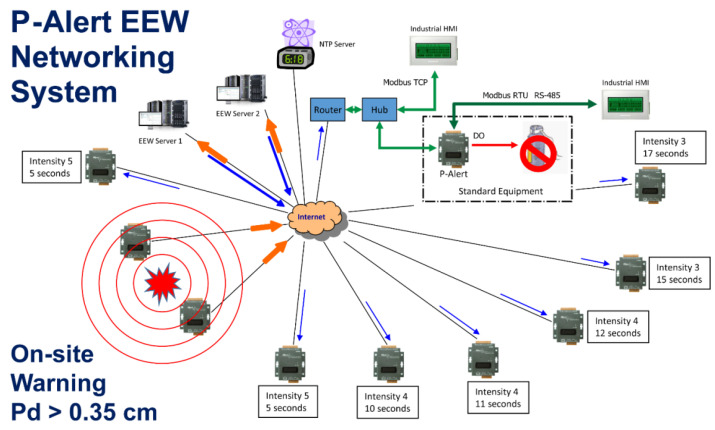
The concept of the P-Alert networking system. Each P-Alert is equipped with the industrial protocol for connection and with two relays that can turn the device on/off for emergency operation. The central station also can turn remote devices on/off through P-Alert relays.

In addition to the on-site warning data used at each station, the data from each field station, received at the central station, is processed continuously for the estimation of threshold parameters ($P_{d}$ and PGA), as well as the magnitude and other parameters, using Earthworm software [4]. Figure 4 shows the setup of P-Alert instruments and the networking scheme of each of the field instruments to the central recording station. Each P-Alert instrument is equipped with the industrial protocol for connection and with two relays that it can switch the device on/off during emergency operations for on-site EEW purposes. The central recording station can also turn the instruments installed in the field on/off through the relay. The warning time in the regional warning is a function of data transmission and epicentral distance. Using a denser recording network, the warning time is maximized by recording the earthquakes promptly. For earthquakes occurring outside of or near the edge of the seismic network, a considerable error is reported in earthquake location and magnitude, and subsequently, the warning is delayed. As the P-Alert instruments are installed densely (5–10 km), the location and magnitude errors are minimized for in-land earthquakes. Even for the earthquakes occurring off the coast of Taiwan, the reported error is manageable. As the P-Alert instruments are installed in various elementary schools, the data flow is continuous from field stations.

## 4. Shakemaps Using P-Alert Network

The NTU network can generate near-real-time shakemaps during earthquakes. A shakemap is a contour demonstrating the PGA, or any other ground-motion parameter distributions, recorded from different strong-motion stations. A seismic network with closely spaced instruments will deliver these shakemaps precisely, as no interpolation is required. Once five P-Alert instruments record a PGA ≥ 1.5 gal, the network starts plotting shakemaps [36]. These shakemaps are updated at regular intervals after 30 s and are delivered to the intended users, including the National Science and Technology Center for Disaster Reduction (NCDR), for damage assessment and possible rescue operations. The shakemaps are also posted on social media, including Facebook and Twitter. With P-Alert instruments distributed all over the country, the shakemaps produced using this instrumentation offer detailed shaking patterns, which are helpful for assessing the damage pattern. The ability to provide shakemaps and to determine rupture direction using this instrumentation is discussed previously in many studies [37,38,39,40,41,42,43].

**Figure 5:**
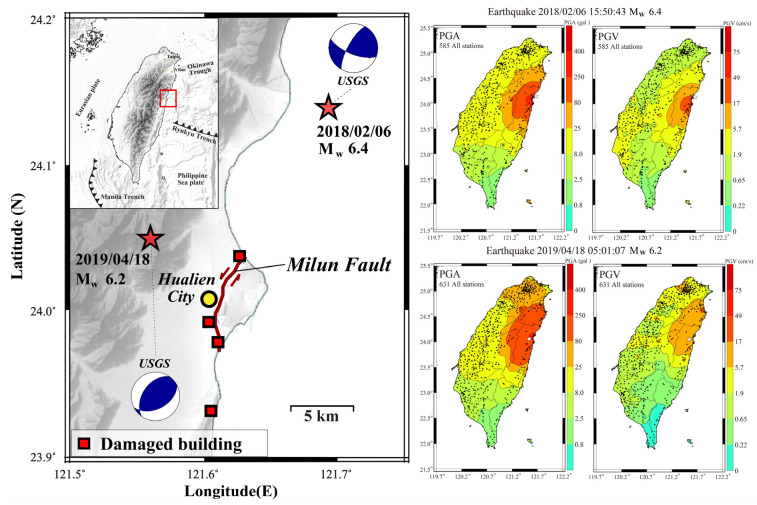
Due to the dense array in operation, real-time shakemaps are plotted. The plotted real-time PGA and PGV Shakemaps for two earthquakes that occurred in the Hualien region.

Yang et al. (2021) [36] upgraded the NTU network to plot additional PGV, CWB Intensity Scale, and $S_{a}$ shakemaps at different periods as value-added products since 2018. The performance of the system with additional shakemaps was checked using the latest recorded earthquakes in the country. With the upgraded system, all shakemaps are now posted on social media after the occurrence of an earthquake. Sometimes, plotted PGV shakemaps have an advantage over PGA shakemaps. For example, Figure 5 shows the plotted PGA and PGV shakemaps during two earthquakes of 2018 and 2019 that occurred in the Hualien region. Mittal et al. (2021) [44] compared the performance of the P-Alert network using plotted shakemaps for these two earthquakes, which had an almost-similar magnitude ($M_{w}$ 6.4 and $M_{w}$ 6.2). The performance was checked in terms of shakemaps. The instruments placed in the epicentral region recorded higher PGA values during both events. The 2018 earthquake had a magnitude $M_{L}$ 6.2 ($M_{L}\;$ reported by the CWB) and caused destruction; however, the 2019 earthquake that had $M_{L}$ 6.3 did not cause any severe damage. From the analysis of PGV shakemaps, a different pattern was observed as compared to PGA. The higher PGV values (>17 cm/s) were observed during the 2018 earthquake, whereas this higher PGV value was recorded by only one instrument during the 2019 earthquake. The damaged areas (buildings suffering collapse and fatalities) during the 2018 earthquake were in the higher PGV areas. Based on the results, it was concluded that PGV may be a better indicator of damage distribution.

## 5. P-Alert Performance during Recent Damaging Events

**Figure 6:**
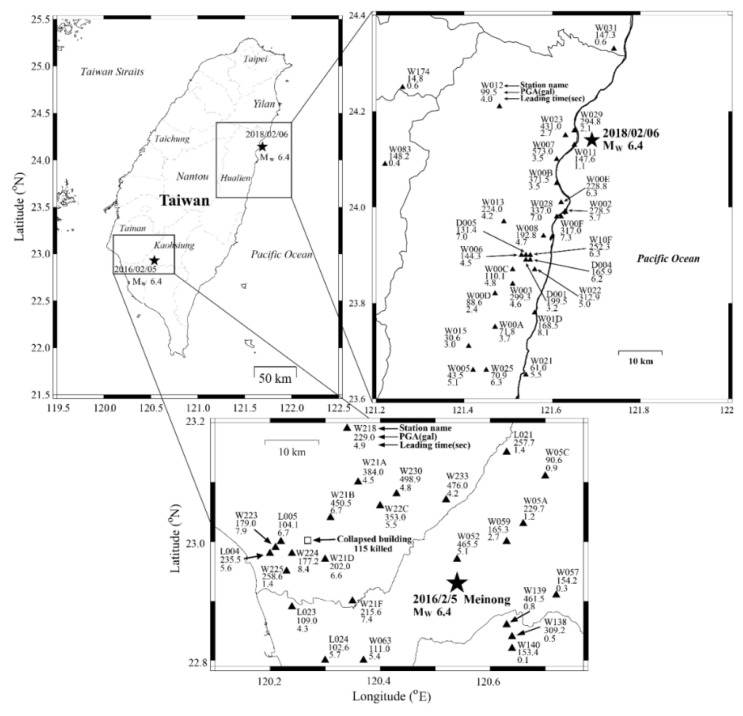
The on-site method can give warnings in the epicentral region where no regional warning is possible. P-Alert records from two earthquakes in 2016 and 2018 show the same trend as Wu and Kanamori (2008) [24] in Figure 2.

The NTU network has shown its ability to work as an on-site EEW system and provide near-real-time shakemaps during recent events. Hsieh et al. (2014) [29] discussed the performance of the P-Alert network during the two earthquakes of 27 March ($M_{L}$ 6.1) and 2 June ($M_{L}$ 6.3) 2013 that occurred in central Taiwan. The working of this network during the Meinong earthquake of 5 February 2016 was discussed by Wu et al. (2016) [17]. The Meinong earthquake was an inland earthquake that occurred in Southern Taiwan and caused more than 117 fatalities. A detailed shakemap was generated by the NTU network within two minutes of the earthquake’s occurrence and the high shaking regions observed in the maps agreed with the damage locations. The individual instrument also provided 4–8 s of on-site warning time before PGA arrival (Figure 6), which is crucial for the locations situated in the blind zone of regional warning. The instruments recorded high PGA values (497 gals) in the epicentral region. Using PGA and PGV shakemaps, it was observed that the highly damaged areas were in high PGV (>17 cm/s) regions.

The Hualien earthquake of 6 February 2018 caused widespread damage in the eastern part of the country. The earthquake caused strong shaking and severe damage to many buildings in Hualien. Lead-times of 1.5–8 s before the arrival of PGA (Figure 6) were obtained in the blind zone at different locations [18]. The PGA during this earthquake, recorded by the P-Alert network, reached around 600 gals, equivalent to a maximum intensity of VII. The PGV recorded by the P-Alert network reached around 125 cm/s without any interruptions [44], showing the robustness of the P-Alert network. By analyzing the P-Alert data of the 2018 Hualien earthquake, it was observed that the data recorded by this instrumentation could be used for surface-wave inversion [18]. Figure 6 shows the on-site warning time generated by different instruments during the 2016 Meinong earthquake and the 2018 Hualien earthquake. During both earthquakes, a useful lead-time is obtained in the blind zone of the CWB regional warning.

## 6. Applications of P-Alert Networks

**Figure 7:**
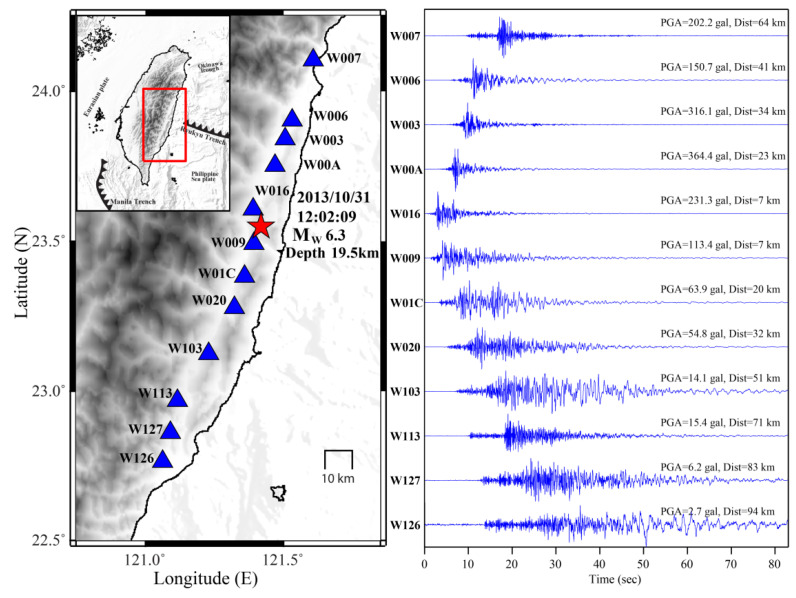
Evaluated fault rupture direction using the recorded waveforms during the 2013 Hualien earthquake. The rupture direction agrees with the aftershock distribution.

Rupture direction is the key parameter and can cause severe destruction, as the ground motion is amplified because of a piling-up of ground motion from near- and far-end instruments. The dense shakemaps generated using P-Alert instrumentation can be used to assess the rupture direction, which is one of the key factors in studying the damage pattern after an earthquake. Wu et al. (2016) [17] found that the rupture direction evaluated using shakemaps during the Meinong earthquake of 2016 agreed with aftershock distribution, which is a usual way of assessing rupture direction. The timely information of rupture direction can help save a lot of lives. The rupture direction using shakemaps from this network during the 2018 Hualien earthquake correlated well with aftershock distribution and surface ruptures [18], which again emphasizes the ability of this instrumentation in estimating rupture direction. Using real-time shakemap interpolation and attenuation regression, Jan et al. (2018) [45] tested the feasibility of using rupture direction from the near-source P-Alert instruments for delivering a warning to the far areas. They used 16 moderate-to-large earthquakes to infer that directivity can be obtained precisely within 17 s of the occurrence of an earthquake, which in turn is very helpful for EEW. Figure 7 shows the rupture direction evaluated using the recorded PGA at selected stations during the Hualien $M_{w}$ 6.3 earthquake of 2013. From the figure, the rupture direction is northeast–southwest and agrees well with aftershock distribution [37].

**Figure 8:**
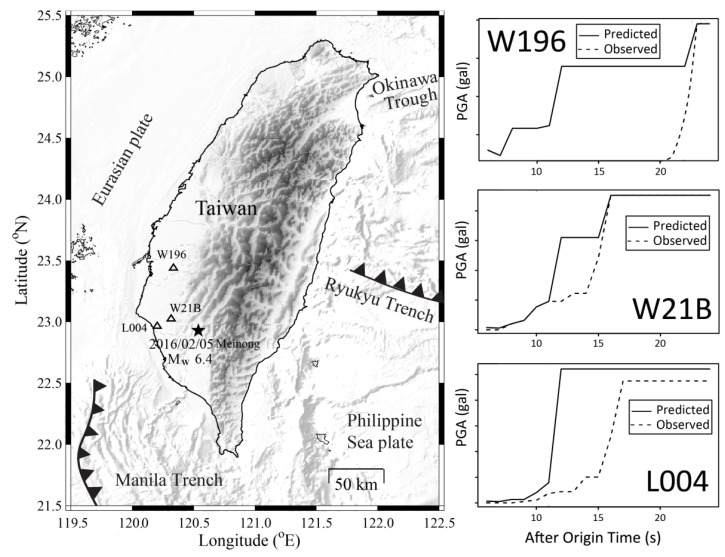
Predicted (solid) and observed (dash) PGA of three P-Alert stations of the 2016 Meinong earthquake. The predicted PGA by the ShakingAlarm approach. Stations W196, W21B, and L004 are 63, 27, and 35 km away from the epicenter, respectively.

The near-real-time detailed shakemaps can identify the direction of the source rupture. Yang et al. (2018) [43] proposed a nontraditional regional EEW system based on time-dependent anisotropic PGA attenuation relationships that are based on real-time P-Alert signals, named “ShakingAlarm”. This is a ground-motion-driven approach using observed data from the source region to establish time-dependent anisotropic PGA attenuation and accurately predict the PGA for the far region before the arrival of the observed PGA. Figure 8 shows the example of the 2016 Meinong earthquake. The stations outside the epicentral region could have had 5–10 s lead-time between the predicted and the observed PGA. The benefit of the ShakingAlarm approach is that it can reliably predict shaking intensity and avoid false alarms, unlike traditional regional EEW systems. However, it cannot provide as long a lead-time as traditional systems do. This is a trade-off problem with EEW, between reliable information and longer lead-times.

Many multi-story buildings in different parts of the world need damage assessments post-earthquakes. The P-Alert instruments have proven efficient in structural health monitoring. Putting a minimum of only three of these instruments can accurately predict the structural health of any building after any earthquake. Hsu et al. (2018) [46] used P-Alert instruments and conducted several shake-table tests with incremental damage to check the performance of P-Alert for evaluating post-earthquake building safety. They found that acceptable damage detection for an entire building is possible using these instruments. The tests were conducted using three types of instruments. It was found that around 50% of P-Alert instruments were correct in identifying the damage level of each story of a building. However, when they aimed to detect the damage to the whole building, these devices displayed 100% correct results. It was concluded that, although P-Alert systems may be a poor indicator of damage location, they correctly depict the damage to an entire building.

Jan et al. (2017) [47] used real-time P-Alert data to determine the coseismic deformation ($C_{d}$) in the epicentral region of earthquakes. The finite-fault model, a crucial component in seismic risk mitigation, can be directly derived from the $C_{d}$ values. They compared the P-Alert $C_{d}$ values with the $C_{d}$ values estimated using Global Navigation Satellite System (GNSS) data and strong-motion data from the Taiwan Strong Motion Instrumentation Program (TSMIP), and found that $C_{d}$ values estimated using the P-Alert network provided useful results, especially for earthquakes having PGA > 60 gals. High $C_{d}$ values ($C_{d}$ > 2 cm) in the epicentral region can help authorities mitigate the damage and act promptly for rescue purposes. High $C_{d}$ values of the order of 60 cm were estimated using the records of the P-Alert network during the 2018 Hualien earthquake and agreed with $C_{d}$ values estimated using GNSS and TSMIP strong-motion data [47].

## 7. P-Alert Worldwide

In several countries, such as China, India, Nepal, Bhutan, Philippines, Indonesia, Greece, Vietnam, the Solomon Islands, and New Zealand, the P-Alert instruments are in-demand because of their low cost and their capability to capture and timely report an earthquake for EEW. All these countries have built EEW networks by either installing P-Alert instruments solely or including them in their existing network. The P-Alert instruments are also popular because they act as two-fold sensors, and separate sensors are not required for on-site and regional warning systems. P-Alert instruments are especially helpful for the countries sitting at the plate boundaries and facing seismic hazards from a large portion (India, China, and Indonesia, to name a few). The embedded algorithms in P-Alert and its effectiveness make it unique; thus, around 3500 P-Alert instruments are already installed around the world.

China established a prototype EEW system in the region of the 1976 Tangshan earthquake using 100 P-Alert sensors since 2010. They also tested the functioning of the EEW system in the Sichuan–Yunnan border region using 270 MEMS sensors, including 100 P-Alert sensors. The functioning of this system is reported by Peng et al. (2019) [48]. India developed its EEW in the Uttarakhand region of the northwest Himalayas by installing 200 P-Alert and P-Alert plus instruments [11] and is currently in the testing stage. This EEW system is planned to issue warnings to the plains regions, including the national capital city, Delhi, from earthquakes occurring around 250–300 km away. Since its installation, no bigger earthquake has been recorded by the network, for which warnings should be disseminated, so to date, no warnings have been issued. In the absence of data from this network, Mittal et al. (2019b) [49] tested the functioning of this EEW network by using the data recorded in Taiwan. They transformed the Taiwan P-Alert stations to match with the Indian stations, and recorded earthquakes within the Indian coordinates. The functioning was tested using the Indian velocity model, the global velocity model, and the Taiwan velocity model. Great accuracy in magnitude and earthquake location was reported using the Indian velocity model. Gujrat State, in India, is also planning to install the P-Alert EEW network for Ahmedabad and other big cities due to the earthquakes occurring in the Kachchh region. The feasibility of working on EEW in this region was put forward by Kumar et al. (2020) [12].

In New Zealand, the P-Alert instruments are distributed by the New Zealand company Jenlogix’s network and are used by several universities, councils, and power companies. The EEW network is specifically designed using these P-Alert instruments.

## 8. Summary and Recommendations

Due to a limited number of installed strong-motion accelerographs at larger distances, the biggest challenge in strong-motion recording is the spatial resolution. A dense network or enough network coverage is the backbone of an EEW system. Because of insufficient station coverage, the estimated earthquake location is error-prone, which, in turn, may cause problems for EEW in terms of estimating strong shaking for the affected areas. For earthquakes occurring at the edge of the network or outside of the network, the error locations may reach 50 km. The seismic arrays have been functional since the 1960s to monitor earthquake activities and to increase signal-to-noise ratios. Despite the advantage of these seismic arrays over regular networks, these are not common in many countries because of the huge cost of instruments, as the instruments are needed at intervals of very few kilometers. The increased spatial resolution in strong-motion recording and the employment of dense networks can be achieved by employing MEMS sensors.

With increased computation power and internet technology, MEMS sensors have proven useful for EEW and other applications. The applicability of MEMS-based sensors has been explored in various countries for EEW. The Quake-Catcher Network formed a seismological instrumentation network by using various MEMS accelerometers [50]. Kong et al. (2016) [51] used real-time MEMS records from smartphones to develop an application called MyShake for EEW. A machine-learning algorithm was used to differentiate between earthquakes and other sources and it was found that estimated earthquake locations and magnitudes were reasonable. Low-cost sensors have demonstrated value in nations, specifically China and India, that confront seismic hazards in a vast region [48]. Cascone et al. (2021) [52] tested the performance of a MEMS sensor prototype in Italy, designed by the Italian company ADEL Srl, to monitor small local events.

The MEMS-based P-Alert sensors have proven helpful in placing the sensors closely together and monitoring the seismic activities minutely. These sensors are cost-effective, low-power consuming, and easy to install. The recent version of P-Alert is promising in terms of dynamic range and storage, the two features that were missing in the earlier version of P-Alert. The main development in the P-Alert network is the continuous increase in the number of stations installed throughout the country. The density and coverage of the instruments are enough, except in the eastern part of the country. A continuous endeavor is being considered to find the proper logistics for the installation of these instruments there. Smartphone-based technology may be another option in Taiwan. In addition to EEW, the P-Alert sensors are used to study the directivity effect, structural health monitoring, and various seismological studies. The low cost of these P-Alert devices has attracted various countries to build their EEW network using these low-cost sensors, or by adding them to their existing network to increase density and network coverage. The results obtained using recorded or real-time data are encouraging and have shown their potential in various applications.

## Data Availability

The strong motion waveform records from the P-Alert network used in this study can be downloaded at http://palert.earth.sinica.edu.tw/db/ (accessed on 15 September 2021).
